# No Evidence of Murine Leukemia Virus-Related Viruses in Live Attenuated Human Vaccines

**DOI:** 10.1371/journal.pone.0029223

**Published:** 2011-12-22

**Authors:** William M. Switzer, HaoQiang Zheng, Graham Simmons, Yanchen Zhou, Shaohua Tang, Anupama Shankar, Beatrix Kapusinszky, Eric L. Delwart, Walid Heneine

**Affiliations:** 1 Laboratory Branch, Division of HIV/AIDS Prevention, National Center for HIV/AIDS, Viral Hepatitis, STD, and TB Prevention, Centers for Disease Control and Prevention, Atlanta, Georgia, United States of America; 2 Blood Systems Research Institute and Department of Laboratory Medicine, University of California (UCSF), San Francisco, San Francisco, California, United States of America; INSERM, France

## Abstract

**Background:**

The association of xenotropic murine leukemia virus (MLV)-related virus (XMRV) in prostate cancer and chronic fatigue syndrome reported in previous studies remains controversial as these results have been questioned by recent data. Nonetheless, concerns have been raised regarding contamination of human vaccines as a possible source of introduction of XMRV and MLV into human populations. To address this possibility, we tested eight live attenuated human vaccines using generic PCR for XMRV and MLV sequences. Viral metagenomics using deep sequencing was also done to identify the possibility of other adventitious agents.

**Results:**

All eight live attenuated vaccines, including Japanese encephalitis virus (JEV) (SA-14-14-2), varicella (Varivax), measles, mumps, and rubella (MMR-II), measles (Attenuvax), rubella (Meruvax-II), rotavirus (Rotateq and Rotarix), and yellow fever virus were negative for XMRV and highly related MLV sequences. However, residual hamster DNA, but not RNA, containing novel endogenous gammaretrovirus sequences was detected in the JEV vaccine using PCR. Metagenomics analysis did not detect any adventitious viral sequences of public health concern. Intracisternal A particle sequences closest to those present in Syrian hamsters and not mice were also detected in the JEV SA-14-14-2 vaccine. Combined, these results are consistent with the production of the JEV vaccine in Syrian hamster cells.

**Conclusions:**

We found no evidence of XMRV and MLV in eight live attenuated human vaccines further supporting the safety of these vaccines. Our findings suggest that vaccines are an unlikely source of XMRV and MLV exposure in humans and are consistent with the mounting evidence on the absence of these viruses in humans.

## Introduction

A gammaretrovirus, called the xenotropic murine leukemia virus (MLV)-related virus (XMRV), has been reported in persons with prostate cancer (PC), chronic fatigue syndrome (CFS), and blood donors with PCR prevalences up to 67% [1], [2]. A related MLV was also reported in about 86% of CFS persons in a separate study [3]. The finding of XMRV and MLV in humans is controversial as subsequent studies have failed to confirm the initial reports [1], [4], [5], [6], [7], [8], [9], [10]. Recent data showing that XMRV was generated in the laboratory during the passage of a human prostate cancer xenograft in nude mice during the generation of the XMRV-infected prostate cancer cell line 22Rv1 raises further doubts about the association of XMRV with human disease [11]. The origin of MLV in humans has also been questioned by the finding of reagents, human cell lines and specimens that are contaminated with MLV sequences [12], [13], [14], [15], [16], [17], [18]. However, additional studies aimed at defining the prevalence of XMRV and related viruses in humans and their association with diseases using an array of diagnostic tests are currently in progress [19].

MLVs are endogenous gammaretroviruses that constitute about 8–10% of the mouse genome and can cause leukemia, lymphoma, and neurological disorders in mice [20]. XMRV shares about 96% nucleotide identity with MLVs classified as xenotropic and which replicate only in non-mouse cells [1]. Thus, while mice are the likely species origin of MLV-related viruses, exposures that may have led to possible cross-species infections may be diverse, ranging from natural exposure to mice to possible exposures of biologicals, such as vaccines [21]. Mice and other rodents have been, or are currently used, in the production of vaccines. For example, the first live polio vaccine was grown in mice and tested on humans in 1950, smallpox, yellow fever, and rabies viruses were cultured in the brains of mice for vaccine production and several live, attenuated vaccines are produced on mammalian cell lines from mice, pigs, chickens, and cats [22]. Thus, MLV may have been introduced into vaccines during attenuation of the master seed stock during successive passage in rodents or during vaccine production from contaminated reagents or growth in mouse or other rodent cell lines.

The Japanese encephalitis virus (JEV) vaccine (SA14-14-2, Rongsheng, China) is an example of a live vaccine that was attenuated via passage in rodents, including mouse brain, primary hamster kidney (PHK) cells, mouse spleen and skin, Syrian hamster (*Mesocricetus auratus*) spleens, and suckling mice skin [23]. Production of the SA-14-14-2 vaccine is done in the hamster PHK cell line. According to the manufacturer, the master seed virus of the SA-14-14-2 JEV vaccine and the PHK cell lines used for vaccine production were shown to be free of adventitious agents and pathogens. The SA-14-14-2 JEV vaccine has been used for over 20 years and administered to over 300 million children in China, South Korea, Nepal, and India.

Although the master seeds and cell substrates used for vaccine production are prescreened for adventitious agents, newer technologies, including sequence-independent amplification, followed by ultra-deep DNA sequencing, and microarrays have shown that some cell substrates and live-attenuated vaccines still contain adventitious viruses, including endogenous retroviruses like avian leukosis virus (ALV), and porcine circovirus [24]. Previous studies have also documented the presence of endogenous avian retroviruses in currently used avian cell-derived vaccines [25], [26]. Endogenous retroviruses exist in all mammals as proviral DNA integrated in the germ line of the host and are passed from parent to offspring. Thus, endogenous retroviruses cannot be eliminated from cell lines or live animals by pre-screening. While most endogenous retroviruses are replication defective, some exist as intact genomes that are capable of expressing infectious virus.

We screened eight live attenuated human vaccines for XMRV and related MLV and adventitious agents using PCR and metagenomics. The eight vaccines included JEV (SA-14-14-2), varicella (Varivax), measles, mumps, and rubella (MMR-II), measles (Attenuvax), rubella (Meruvax-II), rotavirus (Rotateq and Rotarix), and yellow fever virus. All eight vaccines were negative for XMRV and closely related MLV sequences using these two approaches. We found novel hamster genomic and retrovirus sequences in the JEV vaccine mostly likely originating from vaccine production in Syrian hamster cells. Our findings do not support the hypothesis that vaccines are a possible source of XMRV or MLV introduction into humans and are consistent with accumulating evidence on the absence of these viruses in humans.

## Results

### Absence of MLV and XMRV sequences in live attenuated vaccines by PCR testing

**Figure 1:**
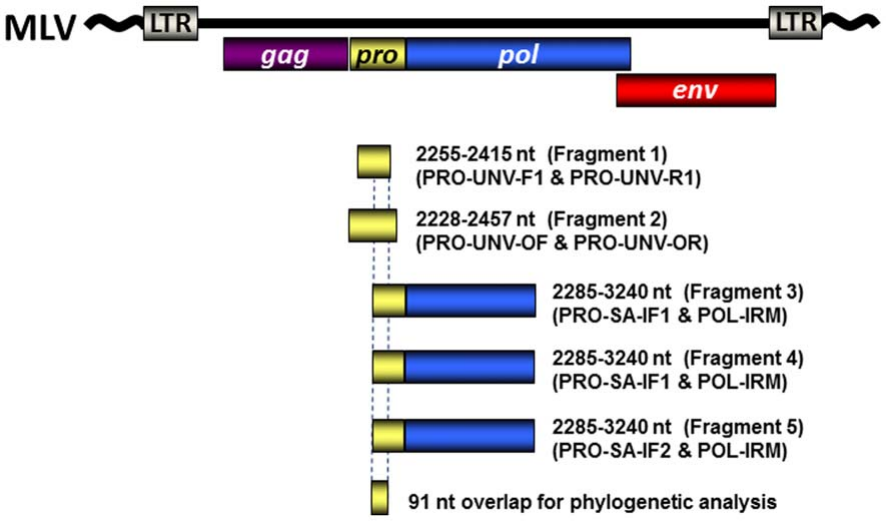
Schematic showing locations and sizes of endogenous retrovirus sequences detected in the live, attenuated Japanese encephalitis virus (JEV) vaccine (SA-14-14-2) and in hamster cell lines. Primer positions are relative to an alignment of prototypical murine leukemia viruses (MLV). Primer names and PCR fragments obtained are provided in parentheses. LTR, long terminal repeat; *gag*, group specific antigen; *pro*, protease; *pol*, polymerase; *env*, envelope. Also shown is the 91-bp overlap region used for phylogenetic analysis of all PCR-amplified fragements. Quantitative PCR with the Taqman primers Pro-UNV-F1 and Pro-UNV-R1 were used to originally detect gammaretroviruses in the JEV vaccine and hamster cell line DNA (fragment 1, 161-bp). Four additional primer sets were used to amplify larger gammaretrovirus sequences for further genetic characterization (Fragments 2–5). Nested PCR was used for obtaining fragments 3–5 and all amplified sequences were about 950-bp in length. Only the inner most primer name combinations are shown. The primers PRO-UNV-OF 5′TAGGGAGGTC AGGGTCAGGAGC3′ and PRO-UNV-OR 5′GGAAAGAGTGRGTGACCT TACCGGT3′ were used to amplify a second fragment (283-bp). The primary PCR amplification for fragment 3 used the primers PRO-UNV-F1 and XPOLIR 5′AAGTGGCGG CCAGCAGTAAGTCAT3′, while the outer primers for fragments 4 and 5 were PRO-UNV-OF and XPOLIR. The internal primers for fragments 3 and 4 were PRO-SA-IF1 5′GAGCAACC AGTCACCTTCCTA3′ and POL-IRM 5′TCTGGGTGCTGGATCCGGAA3′, while the internal primers for fragment 5 were PRO-UNV-IF2 5′ GGGCGCCCRGTCACCTTCCTG3′ and POLIRM.

Total nucleic acids and particle-associated RNA from eight live attenuated vaccines were tested for XMRV and MLV sequences using a generic polymerase (*pol*) and a specific (*gag*) nested PCR test capable of detecting at least 10 DNA and 100 RNA sequences per reaction [2], [3], [27]. In addition, we used a new generic, quantitative real-time PCR (qPCR) test capable of detecting protease (*pro*) sequences of all MLV and XMRV with a reported sensitivity of 10 copies per reaction (fragment 1, Fig. 1) [28]. All eight vaccines were negative for XMRV and MLV DNA and RNA sequences using this combination of PCR tests, except the JEV vaccine which was estimated to contain about 960 copies of MLV-like DNA sequences/ml by using the q*pro* test (Tables 1 and 2). The MLV-like DNA sequences detected in the JEV vaccine by qPCR were confirmed by gel electrophoresis and sequence analysis. However, the amount of MLV-like sequences in the q*pro* amplification product appeared greater in the gel image than that quantified by qPCR when compared to the qpCR assay standards (data not shown). These results indicated detection of a variant with some level of sequence divergence from the generic MLV probes used in the qPCR assay. Indeed, analysis of this 91-bp *pro* sequence identified a distinct gammaretrovirus that is equidistant from murine endogenous retroviruses (mERVs), XMRV, and MLV sharing only 72–75% nucleotide and amino acid identity (data not shown).

**Table 1:** Identification of Hamster DNA and Retroviruses in Eight Live, Attenuated Human Virus Vaccines.

Vaccine	Virus	Manufacturer	Lot Nos.	Virus Preparation	Animal Passage	Metagenomics analysis (non-vaccine viruses)^1^	XMRV/MLV PCR	Murine DNA PCR
SA-14-2-2	Japanese encephalitis	Rongsheng	201002A014-3200906A087-1	live, attenuated	Primary hamster kidney cells, mice, hamsters, primary chick embryo	Autographa c. nucleopolyhedrous virus, HERV-H	Pos^2^	Syrian hamster DNA
Varivax	varicella	Merck	1526X	live, attenuated	embryonic guinea pig	None	Neg	NT^3^
MMR-II	measles, mumps, rubella	Merck	1732X	live, attenuated	chick embryo cell culture	None	Neg	NT
Attenuvax	measles	Merck	1440X	live, attenuated	chick embryo cell culture	avian leukosis virus	Neg	NT
Meruvax-II	rubella	Merck	1198X	live, attenuated	none	None	Neg	NT
RotaTeq	rotavirus	Merck	1724X	live, attenuated	MA104 (rhesus macaque kidney), Vero cells (AGM kidney)	None	Neg	NT
Rotarix	rotavirus	GlaxoSmithKline	A41FA799A	live, attenuated	Vero cells (AGM kidney)	porcine circovirus	Neg	NT
Yellow Fever	yellow fever virus	Sanofi Pasteur	UF430AA-5188	live, attenuated	embryonated chicken eggs	None	Neg	NT

1 Metagenomic results for vaccines other than JEV were reported previously [24].

2 Distinct gammaretrovirus sequences were obtained that were equidistant from MLV and XMRV.

3 Not tested.

**Table 2:** Detection of Japanese encephalitis virus (JEV) RNA, hamster endogenous retrovirus (ERV) DNA, and hamster genomic DNA in the live, attenuated JEV vaccine (SA-14-14-2).

	JEV SA-14-14-2	
PCR Assay	Nuclease^1^−(copies/ml)	Nuclease+(copies/ml)
JEV RNA	4×10^5^	4×10^5^
Murine mtDNA (MCOX2)	negative	negative
IAP-pol DNA^2^		
(Syrian hamster + murine specific)	6.32×10^10^	1.94×10^4^
IAP-pol DNA		
(Chinese hamster specific)	negative	negative
MLV RNA (q*pro*)	9.6×10^2^	negative
Hamster ERV RNA (q*pro*-based)^3^	2.4×10^6^	negative
MLV RNA (ext-q*pro*)^4^	positive	negative
Hamster ERV RNA (ext-q*pro*-based)^5^	2.1×10^7^	negative
MLV RNA (q*gag*)	negative	negative
MLV RNA (nested *gag*)	negative	ND^6^
MLV RNA (nested *pol*)	negative	ND

1 Viral filtrates treated with (+) or without (−) DNase and RNase.

2 IAP, intracisternal A particle; *pol*, polymerase.

3 Primers were based on amplicons generated with MLV quantitative protease (q*pro*) assay.

4 ext, extended q*pro* assay and is equivalent to Fragment 2 in Fig. 1.

5 Primers were based on amplicons generated with extended q*pro* assay.

6 ND, not done.

### Detection and characterization of novel hamster retrovirus and genomic sequences in JEV vaccines

**Figure 2:**
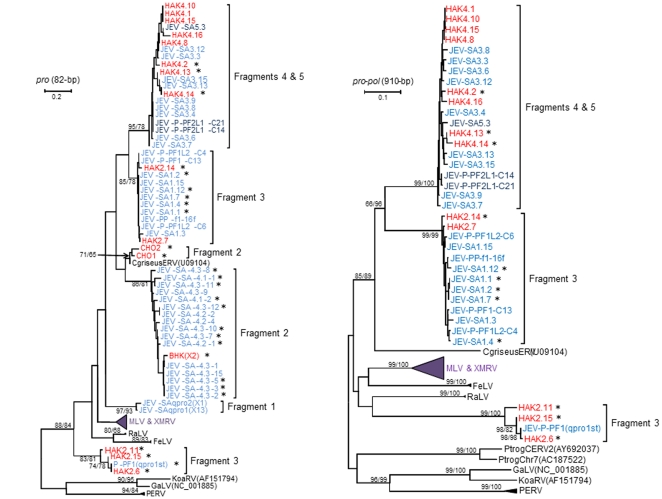
Identification of novel hamster endogenous gammaretrovirus sequences in the live attenuated Japanese encephalitis virus (JEV) vaccine (SA-14-14-2). (a) Phylogenetic inference of 91-bp overlapping (final alignment length is 82-bp) gammaretrovirus DNA sequences PCR-amplified from the JEV SA-14-14-2 vaccine and hamster cell lines (baby hamster kidney (BHK) and Chinese hamster ovary (CHO)) using five different PCR assays. (b) Phylogenetic inference of 910-bp gammaretrovirus DNA sequences PCR-amplified from the JEV SA-14-14-2 vaccine and HAK using three different PCR assays. The new sequences were phylogenetically compared to prototypical gammaretroviruses (GenBank accession numbers in parentheses). MLV, murine leukemia virus; mERV, mouse endogenous retrovirus; FeLV, feline leukemia virus; RaLV, rat leukemia virus; PtrogCERV, Pan troglodytes chimp endogenous retrovirus; GaLV, gibbon ape leukemia virus; KoaRV, koala retrovirus; PERV, porcine endogenous retrovirus. XMRV sequences coded with VP and WPI are from persons with prostate cancer and chronic fatigue syndrome, respectively. X followed by a number in parentheses indicates the number of identical sequences obtained for that fragment. Asterisks indicate sequences without open reading frames. The MLV/XMRV and PERV branches were collapsed to fit the trees to single pages. Stability of the tree topology was tested using 1000 bootstrap replicates in both neighbor joining (NJ) and maximum likelihood (ML) methods. Bootstrap values >60 are shown (NJ/ML). New sequences (fragments 1–4) from the JEV vaccine and hamster cell lines are in light blue and red text, respectively. Fragment 5 sequences amplified from the JEV SA-14-14-2 vaccine are in dark blue text.

To confirm the presence of the distinct gammaretrovirus sequence in the JEV vaccine we designed a new qPCR test specific for the detection and quantification of this sequence. Using this test we estimated that the JEV vaccine contained 2.4×10^6^ copies/ml of this sequence (Table 2). To obtain longer sequences for further viral characterization, we designed additional generic primers flanking the q*pro* primers that are based on a nucleotide alignment of MLV and XMRV complete genomes (fragment 2, Fig. 1). We used these new *pro* primers to test nucleic acids from the JEV vaccine. In addition, we tested DNA from Chinese (Chinese hamster ovary, CHO) and Syrian (baby hamster kidney, BHK) hamster cell lines since the JEV vaccine is produced in hamster cells [23], [29]. Longer *pro* sequences 200–235-bp in length were detected in the JEV vaccine while 235-bp sequences were amplified from the CHO and BHK cell lines, after removal of the primer sequences. BLAST analysis showed that these longer *pro* sequences in the vaccine and BHK were equidistant (∼80% nucleotide identity) from MLVs while those from the CHO cell line were 94% identical to a transcriptionally active endogenous retrovirus (Genbank U09104) found in Chinese hamsters (*Cricetulus griseus*). Interestingly, these 200–235-bp *pro* sequences were distinct from the 91-bp *pro* sequences sharing only 68–75% nucleotide identity. Phylogenetic analysis of 82-bp containing the overlapping regions of the 91-bp and 200–235-bp *pro* sequences confirmed these genetic relationships and showed that these sequences are distinct from MLV and XMRV and were closer in identity to gammaretroviruses in the Chinese (CHO) and Syrian hamster cell lines (BHK and HAK) (Fig. 2a).

We next designed additional forward PCR primers based on the consensus q*pr*o and the 200–235-bp *pro* vaccine sequences and new generic reverse primers based on MLV and XMRV prototype *pro*-*pol* sequences and applied these to the JEV vaccine and hamster cell line nucleic acids to obtain longer and more phylogenetically informative sequences (Fig. 1). A total of three different primer combinations were used (Fig. 1) which generated three different groups of DNA sequences 950-bp in length (fragments 3–5) from the HAK cell line DNA and the JEV vaccine and all were distinct from the 92-bp *pro* sequences. These *pro*-*pol* sequences were cloned and analyzed and determined to be equidistant from other murine retroviruses sharing only 70% nucleotide identity and were 77% identical to each other. Phylogenetic analysis of an alignment containing 910-bp of the 950-bp fragments alone or with the overlapping *pro* regions (82-bp) confirmed that these new *pro*-*pol* sequences are distinct from other gammaretroviruses in rodents and other mammals (Fig. 2). When all five PCR fragments were aligned and analyzed, six different gammaretrovirus phylogenetic lineages were inferred (Fig. 2a). The first lineage included the original qpro (Fragment 1) sequences that were distinct from MLV, XMRV and other gammaretroviruses, including those found in the JEV vaccine and hamster cell lines. The second lineage contained fragment two sequences from the JEV vaccine and Syrian hamster cell line, BHK, while the third lineage only contained gammaretrovirus sequences from the Chinese hamster. Lineage four contained distinct JEV (fragment 3) and Syrian hamster sequences from the cell line HAK, while the fifth lineage consisted of JEV fragment 4 and 5 sequences and distinct Syrian hamster cell line (HAK) gammaretrovirus sequences. The sixth lineage was composed of a divergent JEV sequence using the fragment three PCR primers and a third set of Syrian hamster sequences obtained from the HAK cell line. The sixth lineage was also the most divergent, sharing a common ancestor with MLV/XMRV, RaLV, FeLV, and the other hamster and JEV gammaretrovirus sequences (Fig. 2a).

To further characterize the hamster and JEV gammaretroviruses, we phylogenetically analyzed the larger 910-bp alignment consisting of only the fragment 3 to 5 sequences and the appropriate reference sequences (Fig. 2b). Like the phylogenetic analysis of the shorter 82-bp fragments, two distinct fragment 3 lineages and a single lineage containing the fragment 4 and 5 JEV and hamster sequences were inferred. The two groups of 910-bp *pro*-*pol* sequences from the JEV vaccine and HAK DNA containing fragments 3 to 5 were also distinct from, but clustered with the endogenous retrovirus (Genbank U09104) found in Chinese hamsters (*Cricetulus griseus*) (Fig. 2b). The additional 910-bp *pro*-*pol* fragment 3 sequences clustered more closely with rat leukemia viruses (RaLV) but with moderate bootstrap support (Fig. 2b). All three 910-bp *pro-pol* sequence lineages also contained sequences from the HAK Syrian hamster cell line with strong bootstrap support suggesting they are all of hamster origin (Fig. 2b). 950-bp *pro-pol* sequences were not detected in the BHK and CHO cell line DNAs using these primer combinations. The finding of highly divergent gammaretrovirus sequences using these *pro-pol* primer combinations is consistent with the detection of endogenous retroviruses present in hamster DNA.

We evaluated these new sequences for open reading frames (ORFs) indicative of intact viral genomes with replication potential. The two different, 91-bp q*pro* sequences (fragment 1) and all 950-bp *pro-pol* fragment 4 and 5 sequences in the JEV SA-14-14-2 vaccine contained ORFs. In contrast, a single distinct fragment 3 sequence found in the JEV vaccine and three HAK *pro-pol* fragment 3 sequences (950-bp) that clustered weakly with rat leukemia virus RaLV were all defective. A mixture of cloned sequences with ORFs and defective ORFs were present in the second class of fragment 3 *pro-pol* sequences (950-bp) from the SA-14-14-2 vaccine and HAK cell line (71% and 50%, respectively). Almost 69% of the fragment 2 *pro* sequences in the SA-14-14-2 vaccine and all BHK and CHO sequences were defective. Thus, the majority of the cloned gammaretrovirus sequences detected in the attenuated JEV vaccine and hamster cell lines were defective in the subgenomic regions examined.

To investigate whether the retroviral sequences in the JEV vaccines originated from endogenous proviral elements present in DNA or were particle-associated RNA, we PCR-tested viral filtrates that were first treated with and without nucleases. In the presence of nucleases only JEV viral RNA was detected in the filtrates indicating that the gammaretrovirus sequences in this vaccine were not particle associated. Testing particle-associated RNA using primers specific for the five new classes of gammaretrovirus sequences identified in the vaccine yielded negative results (Table 2) confirming that these sequences are not particle-protected and likely represent genomic DNA from the hamster cell substrate in which the vaccine was grown (Table 1). Furthermore, essentially equivalent amounts of *pro* signals were obtained when vaccine nucleic acids were tested by RT-PCR with and without reverse transcriptase documenting retroviral DNA in these extracts (data not shown). However, the presence of particle-associated JEV RNA in the two vaccines was confirmed by the detection of 4×10^5^ copies/ml JEV in both the untreated and the nuclease-treated SA-14-14-2 vaccine filtrates. The attenuated JEV vaccine was also negative for murine mtDNA and IAP sequences but was positive for Syrian hamster, but not Chinese hamster, IAP sequences (∼4×10^8^ copies/ml) using new real-time PCR tests that detect murine and hamster IAPs (Table 2). These new IAP tests are extremely sensitive for detecting mouse DNA contamination in a specimen and can detect 10 attograms of mouse DNA in a background of 1 ug human DNA (data not shown). Our finding of residual Syrian hamster IAP DNA sequences even after filtration and nuclease treatment of vaccine material demonstrate the sensitivity of this new IAP assay for detecting low levels of IAP DNA. These results most likely reflect incomplete digestion of DNA in the material (Table 2). Combined with the presence of endogenous hamster retrovirus DNA sequences, the IAP results further demonstrate that the JEV SA-14-14-2 vaccine contains residual hamster and not mouse DNA.

### Absence of adventitious agents in attenuated JEV vaccines by metagenomics analysis

We have previously shown by metagenomics analysis that the attenuated Attenuvax measles contained viral particle-associated RNA for ALV and endogenous avian retrovirus (EAV) [24]. Likewise, yellow fever and MMR-II vaccines, also made in chick embryo cells, were previously found to contain particles with ALV and EAV RNA [25], [26]. The rotavirus vaccine Rotarix was shown to contain porcine circovirus 1 (PCV1) [24] while PCR studies showed RotaTeq to contain low levels of PCV1 and PCV2 (http://www.ncbi.nlm.nih.gov/pubmed/21569811). Using viral metagenomics and a BLASTx E score cutoff of 0.001 to any viral sequences we found no evidence of adventitious viruses in the live, attenuated JEV (SA-14-14-2) (Table 1). A total of 1993 pyrosequence reads of over 100 nucleotides in length were generated. These sequences included 48 contigs containing 281 reads that were identified as JEV. One near perfect read was identical to *Autographa californica nucleopolyhedrovirus*, which is a common insect baculovirus sprayed on vegetable fields to control insect pests and therefore most likely represents an environmental contaminant. A 365-bp sequence with 99% nucleotide identity to a human genomic region (GenBank accession no. AJ289710) annotated as the human endogenous retrovirus type H was also present in the JEV vaccine and most likely represents human DNA contamination (Table 1). The inability of the metagenomics analysis to detect the novel gammaretrovirus and hamster IAP sequences reflects that these DNAs are not particle-associated.

## Discussion

Despite the production of most strains of attenuated viruses on primary animal-derived cells, live attenuated vaccines are safe, inexpensive, and highly effective at reducing mortality and morbidity from numerous diseases, and thus are used worldwide. We tested eight live attenuated vaccines used globally to prevent viral diseases. We found no evidence of either XMRV or MLV in all eight vaccines using sensitive PCR testing for both viral RNA and DNA sequences. We also show by ultra-deep sequencing of particle-protected nucleic acids the absence of XMRV and MLV in these vaccines. Thus, our data do not support the hypothesis that these vaccines are a possible source of human exposures to XMRV or MLV. These results are consistent with the production of all these vaccines in non-mouse cells which substantially reduces the risks of adventitious endogenous murine retroviruses.

There are other mouse-derived biological products that may be at a higher risk of carrying MLV, such as monoclonal antibodies (mAbs) that are used in humans for the treatment of cancer, inflammatory, and autoimmune diseases [30]. Indeed, mouse hybridomas that have been shown to be contaminated with endogenous xenotropic MLVs [30], but the production of mouse mAbs for clinical use requires purification steps that inactivate and remove viral particles. Our study is also limited by the testing of only eight vaccines and does not include other live attenuated vaccines that have a history of passage in rodents, such as early versions of the oral polio vaccine. We also did not test master virus seed stocks (MVSS) and master cell banks (MCB) used in the manufacture of the live attenuated vaccines from our study since these materials were not readily available. Nonetheless, our testing of vaccines used on humans was a more direct public health risk assessment for adventitious agents that vaccine recipients may have been exposed to compared to MVSS and MCB. Furthermore, the recent finding that XMRV originated as a laboratory artifact during passaging of a prostate tumor xenograft in inbred mice [11], the ability of human serum to inactivate MLV, and the accumulating evidence from a large number of epidemiologic studies showing the absence of XMRV or MLV in humans, all cast doubts on the links of XMRV or MLV to prostate cancer and CFS and their endemicity in human populations [4], [5], [10], [14], [15], [16], [17], [28], [31], [32], [33], [34]. Likewise, the proposed laboratory origin of XMRV in the 22Rv1 cell line around 1996 suggests this virus could not have contaminated vaccines prior to that date [11].

Although we did not find XMRV or MLV in any of the eight vaccines, our data reveal that the live attenuated JEV vaccine (SA-14-14-2) contains multiple hamster genomic DNA and endogenous gammaretrovirus sequences that are distantly related to MLV. Phylogenetic analysis showed that these sequences most likely originated from Syrian and not Chinese hamsters, which is consistent with the reported production history of the JEV SA-14-14-2 vaccine in primary hamster kidney and Syrian hamster spleen [23]. Importantly, specific PCR showed that these sequences are not particle associated, which is consistent with the metagenomics testing, and likely reflects genomic material present in the hamster substrate cells in which the vaccine is produced. Cell substrate DNA is commonly found in vaccines and is unlikely to cause safety risks. Other live attenuated JEV vaccines that are not produced in rodents are currently in preclinical and clinical trials and have demonstrated good immunogenicity and are apparently safe [29], [35].

### Conclusions

We found no evidence of XMRV or MLV in eight globally used live attenuated vaccines and thus our results do not support the hypothesis that these vaccines contributed to the iatrogenic introduction of XMRV or MLV into the human population and are in agreement with the accumulating evidence on the absence of these viruses in human populations. We identified residual hamster DNA containing multiple endogenous gammaretrovirus sequences, but not retroviral RNA, in the JEV vaccine and show that these sequences are of Syrian hamster origin consistent with the production history of the vaccine.

## Methods

### Vaccines

Single dose, live attenuated SA 14-14-2 (Rongsheng; lot nos. 200906A087-1 and 201002A014-3) JEV vaccines were stored at 4°C and were re-suspended in the supplied 1.0 ml PBS. Additional lyophilized live attenuated vaccines, as previously described [24], were resuspended in 200 µl of manufacturer-appropriate sterile diluent (Merck; lot no. 4089) or sodium chloride solution (Sanofi Pasteur; lot no. UF198AB). Rotarix (rotavirus, GlaxoSmithKline [GSK]; lot no. A41XA799A) and Rotateq (rotavirus; Merck; lot no. 1724X) were resuspended in a 1-dose volume of accompanying oral diluent. A total of 200 µl of Meruvax (rubella; Merck; lot no. 1198X), Attenuvax (measles; Merck, lot no. 1440X), YF-VAX (yellow fever; Sanofi Pasteur; lot no. UF430AA-5188), MMR-II (measles, mumps, rubella; Merck; lot no. 1732X), Rotateq, or Varivax (varicella virus; Merck; lot no. 1526X) was filtered through a 0.45 µM filter (Millipore).

### Viral particle purification and nucleic acid extraction

Vaccines were passed through a 400 nm filter and filtrate containing viral particles was treated with a mixture of DNase and RNase to remove exogenous, unprotected nucleic acids as described previously [24]. Particle-associated nucleic acids were then prepared using a QIAamp viral RNA extraction kit (Qiagen). Nucleic acids were also extracted directly from the JEV vaccine using the QIAamp viral RNA Mini extraction kit (Qiagen).

### Virus-specific PCR for MLV, XMRV, and JEV

Both MLV-generic and XMRV-specific PCR tests were used to screen the vaccine nucleic acids. Nested and real-time PCR tests were used to generically detect MLV and XMRV *gag* and *pro* sequences, respectively. The *gag* PCR test used the primers 419F and 1154R in the primary amplification and GAG-I-F/GAG-I-R for the nested PCR reaction [2], [3], [27]. The *pol* PCR test used the primers XPOL-OF and XPOL-OR in primary and XPOL-IF and XPOL-IR in the nested PCR reactions [27]. The quantitative real-time PCR test in the protease (*pro*) region, called q*pro*, used the Taqman primers Pro-UNV-F1 and Pro-UNV-R1 and probes Pro-UNV-P1C and Pro-UNV-PR1 [28] (Fig. 1). The XMRV and MLV generic *gag* quantitative real-time PCR test was performed with the primers GAG-UNV-F1, 5′AGGTAGGAACCACCTAGTYC3′ and GAG-UNV-R1, 5′GTCCTCAGG GTCATAAGGAG3′ and probes GAG-UNV-P1C, 5′FAMAGCGGGTCTCCAAAACGCGGG C3′BHQ1 and GAG-UNV-PR1, 5′FAMCCTTTTACCTTGGCCAAATTGGTGGG3′BHQ1. JEV RNA was detected using the Taqman primers and probes JE-multi-forward and JE-multi-reverse and Multi-probe, as previously described [36].

### Metagenomics analysis of live, attenuated vaccines

Viral cDNA synthesis and random PCR amplification were performed as previously described [24]. Briefly, 100 pmol of primer consisting of an arbitrarily designed 20-base oligonucleotide followed by a randomized octamer (8N) sequence at the 3′ end was used in a reverse transcription (RT) reaction (Superscript III; Invitrogen). Two distinct primers containing different 20-base fixed sequences were used in two separate RT reactions targeting the virus-enriched nucleic acids from the live-attenuated JEV vaccine. A single round of DNA synthesis was then performed using Klenow fragment polymerase (New England Biolabs), and then PCR amplification of double-stranded DNA using a primer consisting of only the 20 fixed bases was performed. Independent duplicate PCRs were performed for each random primer, generating a total of 4 separate reactions. Random PCR DNA products were pooled and separated on an agarose gel, and fragments from 500 bp to 1,000 bp were excised and extracted. DNA was sequenced using GS FLX Titanium reagents.

### Sequence read classification

The 454 sequence reads were trimmed of their random PCR primer sequences and assembled into longer contigs using the program Sequencher (Genecodes), with an overlap set as 95% similarity over 35-bp to merge fragments. The contigs and singlets greater than 100-bp were compared to the NCBI nonredundant nucleotide and protein databases using BLASTn and BLASTx, respectively. Viral sequences were classified based on their best alignment E value and those with values <10e–3 were deemed unclassifiable.

### Cell line DNA

Syrian baby hamster kidney (BHK), Syrian hamster kidney (HAK), and Chinese hamster ovary (CHO) cryopreserved cell lines were obtained from the CDC Biologics Branch and DNA was extracted using the Qiagen Flexigene DNA kit.

### Identification of novel hamster gammaretrovirus sequences in the JEV vaccines

To further characterize the gammaretrovirus sequences identified in the JEV SA-14-14-2 vaccine additional PCR primers were designed based on an alignment of endogenous murine and hamster retrovirus sequences available at GenBank and the new sequences obtained with the q*pro* primers and subsequent amplicons. The primers PRO-UNV-OF 5′TAGGGAGGTC AGGGTCAGGAGC3′ and PRO-UNV-OR 5′GGAAAGAGTGRGTGACCT TACCGGT3′ were used to amplify a second fragment (283-bp) surrounding the q*pro* sequences from the SA-vaccine and BHK and CHO cell line DNAs (Fig. 1). To increase the sensitivity of detecting divergent murine or hamster gammaretroviruses we used the following nested PCR primer combinations to amplify three additional fragments about 950-bp in size from the JEV SA-14-14-2 vaccine and HAK cell line DNA (Fig. 1). The primary PCR amplification for fragment 3 used the primers PRO-UNV-F1 and XPOLIR 5′AAGTGGCGGCCAGCAGTAAGTCAT3′, while the outer primers for fragments 4 and 5 were PRO-UNV-OF and XPOLIR. The internal primers for fragments 3 and 4 were PRO-SA-IF1 5′GAGCAACCAGTCACCTTCCTA3′ and POL-IRM 5′TCTGGGTGCTGGATCCGGAA3′, while the internal primers for fragment 5 were PRO-UNV-IF2 5′ GGGCGCCCRGTCACCTTCCTG3′ and POLIRM (Fig. 1). The Expand High Fidelity PCR System (Roche) was used for the primary amplification and Amplitaq (ABI) for the nested PCR. 25 ul of extracted nucleic acids or 100 ng cell line DNA were used as templates in the primary PCR amplification. Forty cycles of each round of PCR was done using 95°C for 30 sec, 50°C for 30 sec, and 72°C for 1 min.

### PCR detection of mouse and hamster DNA contamination

Mouse mitochondrial DNA (mtDNA) and intracisternal A particle (IAP) sequences were detected using the primers MCOX2F2 and MCOX2R1 and probes MCOX2PR1 and MCOX2P1 and IAP-F and IAP-R using the conditions previously reported [16], [28]. Given that the JEV SA-14-14-2 vaccine was also passed in hamsters we also designed two new real-time PCR tests that generically detect mouse and Syrian hamster IAP or specifically detect Chinese hamster (CHO) IAP *pol* sequences. The generic IAP primers and probes are IAP-MH-POLF2: 5′ GCCTCAYATGTG ATTCAACATTG 3′ and IAP-MH-POLR2: 5′ TTGRGASGTATAWGCTGGT CCATT 3′ and IAP-MH-POL P2: 5′ FAM TTGAGGCMTGGAGTGCTTGGGGRAAACCCAGA 3′BHQ1, respectively. The CHO-specific IAP primers and probe are IAP-MH-POLF3 5′GCCGCGCATGTGAT TCAACACTG3′, IAP-MH-POLR3 5′ TTGAGAGGTATAAGCCGGCCCAT3′, and IAP-MH-POLP3 FAM5′TAGAGGCTTGGGGTGCCTGGGGTAAACCTCAT3′BHQ1. The assay was performed with a hot start at 95°C for 9 min followed by 55 cycles of PCR at 95°C for 30 sec and 62°C for 30 sec. This assay was 1000X more sensitive in detecting IAP sequences in mouse DNA than that using the IAP-F and IAP-R primers though both and the mtDNA PCR tests could detect 1 copy each in a background of 1 ug of human DNA (data not shown).

### Sequence analysis

PCR products were purified with QiaQuick PCR or gel purification kits (Qiagen, Valencia, CA) and were directly sequenced on both strands by using ABI Prism BigDye terminator kits and an ABI 3130×l sequencer (Foster City, CA) or following cloning in the TOPO vector (Invitrogen). Initial sequence identity was determined using BLAST analysis at the National Center for Biotechnology Information web server (http://blast.ncbi.nlm.nih.gov/Blast.cgi) using either the megablast or blastn search options. Sequences were aligned with those retrieved from the BLAST analysis with the highest nucleotide identity, and other MLV prototypes available at GenBank, using Clustal W in the MEGA v5.03 program (http://megasoftware.net/). Following manual editing and removal of indels, substitution models and phylogenetic relationships were inferred using the neighbor joining (NJ) method implemented in MEGA v5.03. Phylogenies were also inferred using the program PhyML plugin in the Geneious software package v5.3.6 (www.geneious.com) that implements the fast maximum likelihood (ML) method. Support for the branching order was evaluated using 1,000 nonparametric bootstrap replicates.

### Nucleotide sequence accession numbers

The new hamster gammaretrovirus *pro-pol* sequences generated in the current study are available at GenBank with accession numbers JN652837–JN652873. An alignment of the 91-bp *pro* sequences is available from the authors upon request. GenBank does not accept sequences less than 200-bp in length.
